# Supplementary material for: Priming antibody responses to the fusion peptide in rhesus macaques
Source: NPJ Vaccines. 2024 Jul 12;9:126. doi: 10.1038/s41541-024-00918-9 (PMC11245479; doi:10.1038/s41541-024-00918-9)
Supplement: Supplementary file 1 — Supplemental Information [file 41541_2024_918_MOESM1_ESM.pdf]

**Supplemental Information.**

# Priming antibody responses to the fusion peptide in rhesus macaques

**Short Title/ Running Title: Fusion Peptide Targeting in NHP**

Christopher A. Cottrell[1,2,#], Payal P. Pratap[1,2,#], Kimberly M. Cirelli[3], Diane G. Carnathan[2,4], Chiamaka A. Enemuo[4], Aleksandar Antanasijevic[1,2], Gabriel Ozorowski[1,2], Leigh M. Sewall[1,2], Hongmei Gao[5], Joel D. Allen[6], Bartek Nogal[1,2], Murillo Silva[8], Jinal Bhiman[9], Matthias Pauthner[2], Darrell J. Irvine[2,8], David Montefiori[5], Max Crispin[6], Dennis R. Burton[2,7,10], Guido Silvestri[2,4], Shane Crotty[2,3,11], and Andrew B. Ward[1,2]*

## Author Affiliations

[1] Department of Integrative Structural and Computational Biology, The Scripps Research Institute, La Jolla, CA 92037, USA
[2]International AIDS Vaccine Initiative Neutralizing Antibody Center and Scripps Consortium for HIV/AIDS Vaccine Development (CHAVD), The Scripps Research Institute, La Jolla, CA 92037, USA.
[3]La Jolla Institute for Immunology, La Jolla, CA 92037, USA.
[4]Division of Microbiology and Immunology, Emory National Primate Research Center, Emory University, Atlanta, GA 30329, USA.
[5]Duke Human Vaccine Institute and Department of Surgery, Duke University Medical Center Durham, NC, USA.
[6]School of Biological Sciences, University of Southampton, Southampton, SO17 1BJ, UK
[7]Department of Immunology and Microbiology, The Scripps Research Institute, La Jolla, California, USA.
[8]Koch Institute for Integrative Cancer Research, Massachusetts Institute of Technology, Cambridge, MA 02139, USA.
[9]Centre for HIV and STI, National Institute for Communicable Diseases of the National Health Laboratory Service, Johannesburg, South Africa.
[10]Center for HIV/AIDS Vaccine Immunology and Immunogen Discovery and IAVI Neutralizing Antibody Center, Department of Immunology and Microbiology, The Scripps Research Institute, La Jolla, California, USA.
[11]Division of Infectious Disease and Global Public Health, Department of Medicine, University of California, San Diego, La Jolla, California, USA
[*]Correspondence: andrew@scripps.edu
[#]Contributed equally

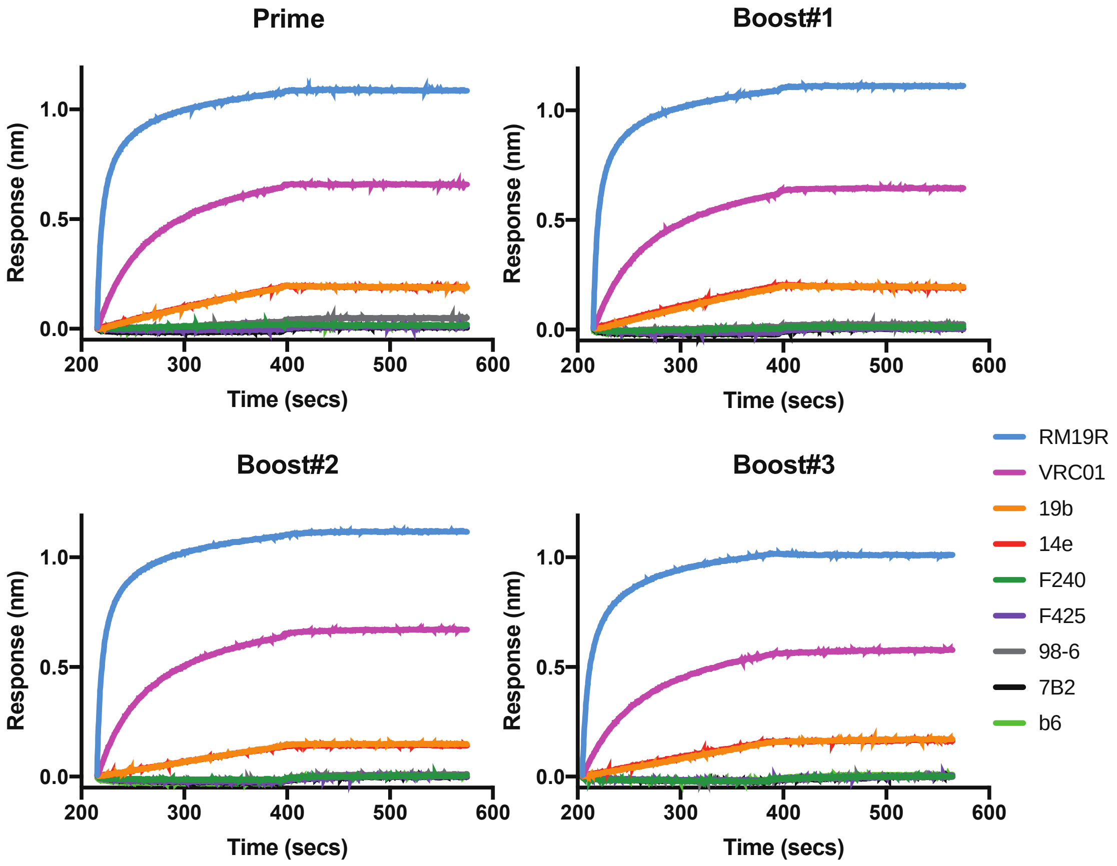


**Supplementary Figure 1. Antigenicity of FP targeting immunogen series.**

RM19R is a base-binding mAb elicited in a BG505 SOSIP.664 immunized RM (*9*). VRC01 is a CD4 binding site targeting bnAb (*34*) and b6 is a CD4 binding site targeting non-neutralizing mAb. 19b, 14e, and F425 are V3 targeting non-neutralizing mAbs. F240, 7B2, and 98-6 post-fusion gp41 targeting mAbs.

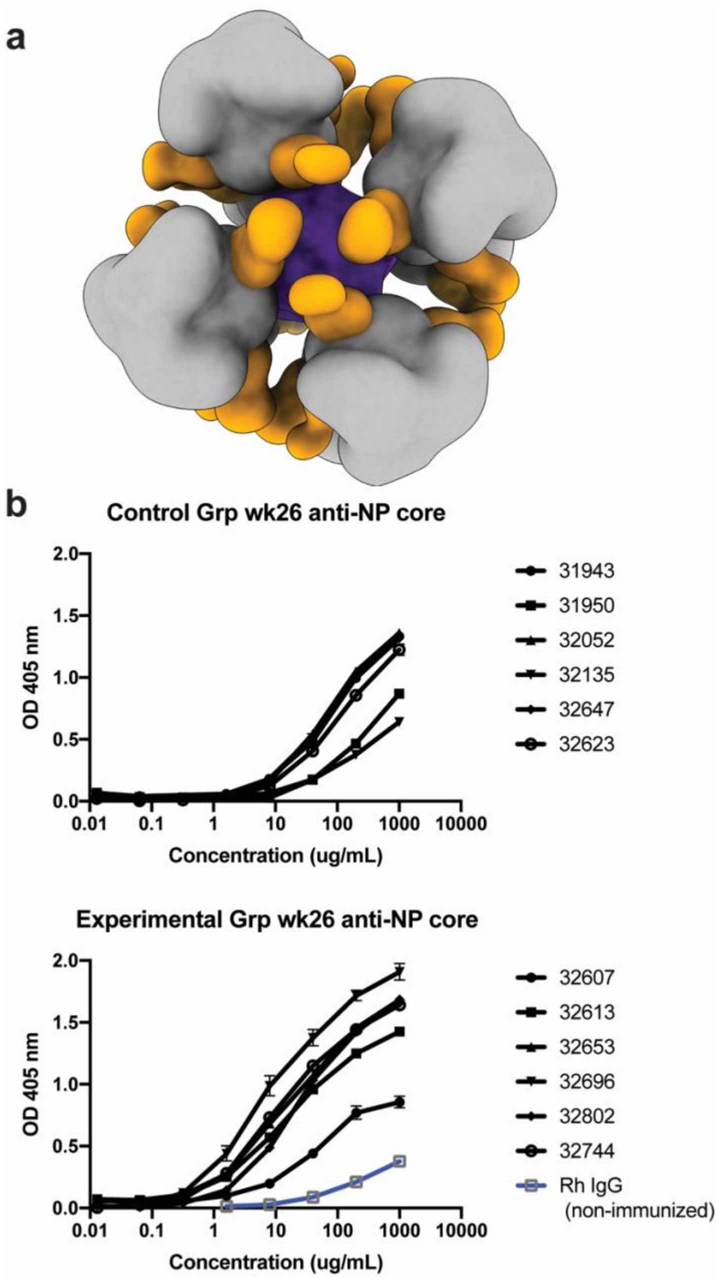


**Supplementary Figure 2. T33-31 nanoparticle core antibody binding.**

A) T33-31 nanoparticle core (purple) displaying Boost # (grey) that is bound by FP bnAb VRC34 fab (orange). B) IgG purified from week 26 was analyzed via ELISA at 5-fold dilutions to determine IgG recognition of T33-31 nanoparticle core. Upper panel shows response for the control group monkeys while lower panel shows response for the experimental group monkeys.

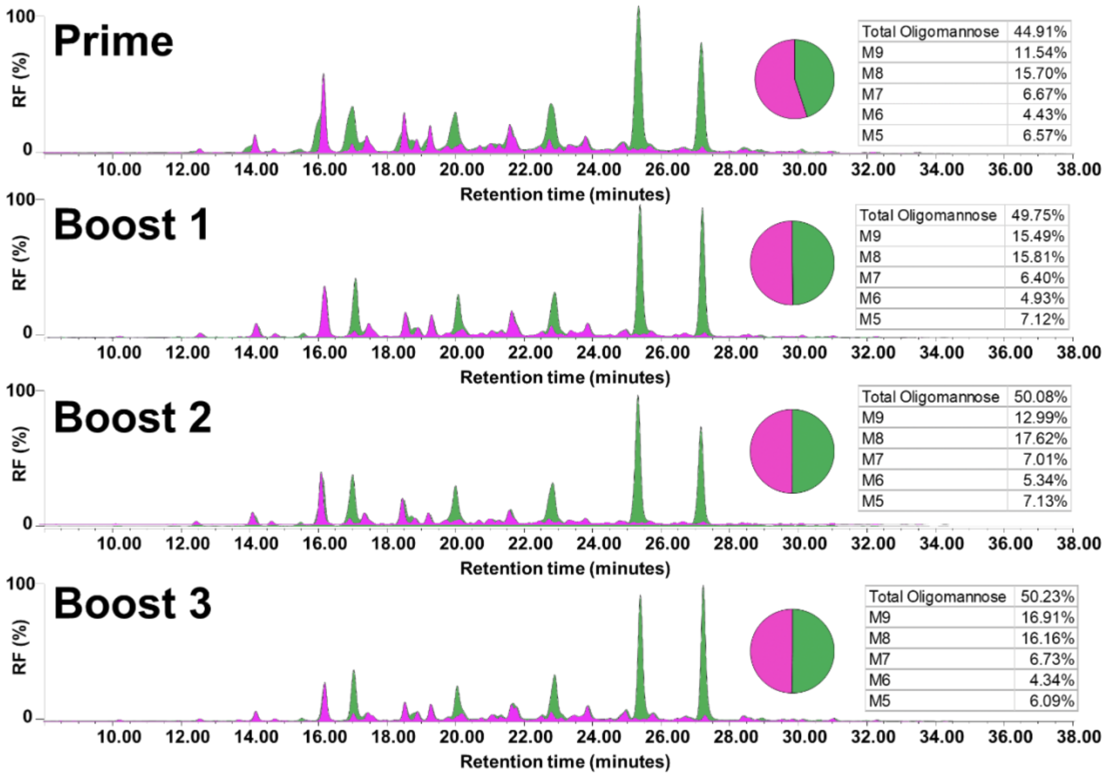


**Supplementary Figure 3. UPLC analysis of released N-glycans from the sequential immunogens.** Procainamide labelled glycans were subjected to endoH digestion that enables the determination and quantification of oligomannose and hybrid-type glycans (green) and complex-type glycans (magenta). Oligomannose-type glycans were quantified using the Waters Empower software.

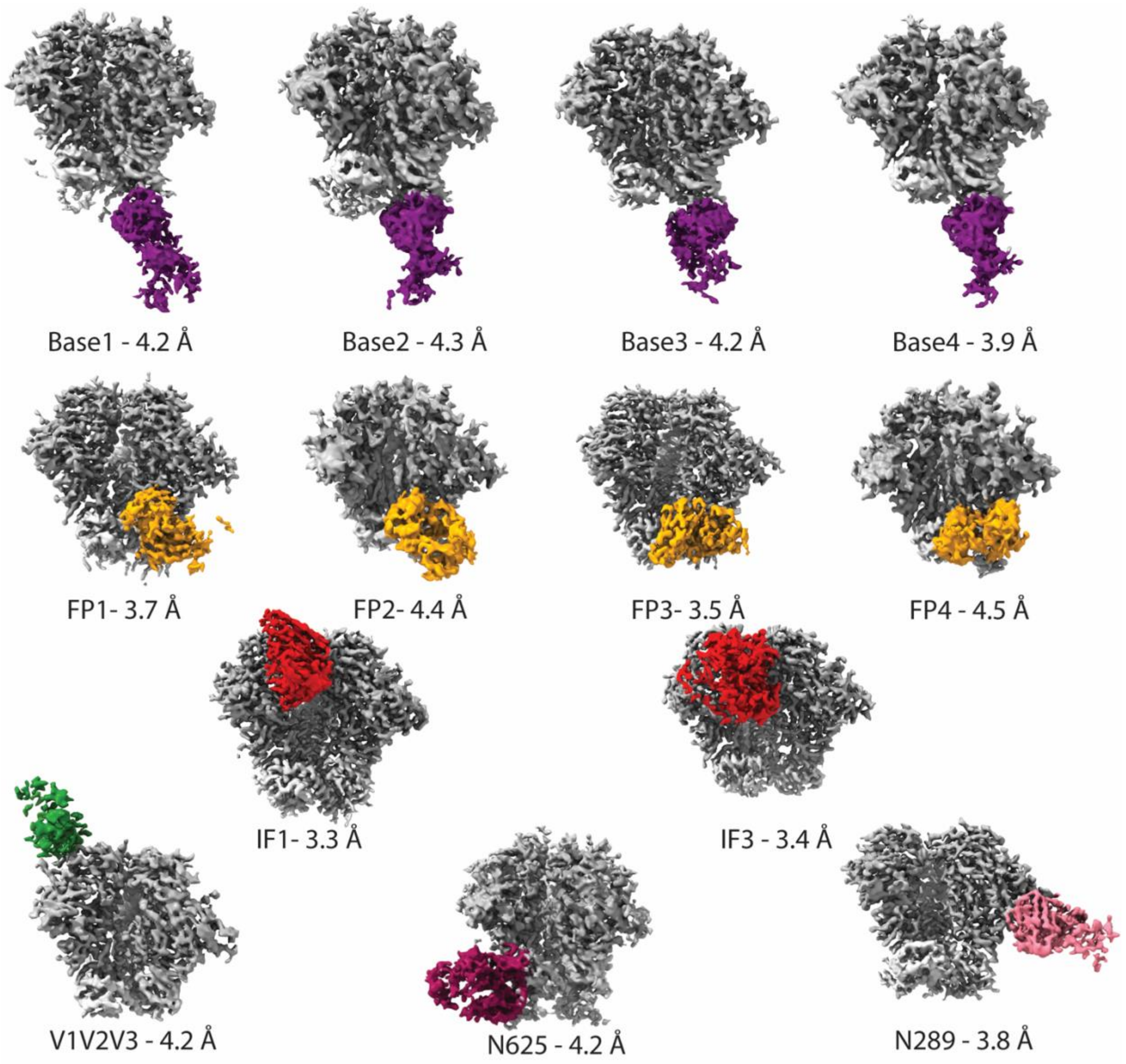


**Supplementary Figure 4. CryoEMPEM High Resolution Maps**

Thirteen high resolution (≤4.5 Å) maps were resolved from one polyclonal sample (animal 32613 at week 42 timepoint). Resolved maps include four against the base of the trimer, four maps against the fusion peptide (FP) region, two against the interface (IF) region, one against the V1V2V3 variable region, one against the N625 gp41 glycan and one against N289 glycan of gp120.

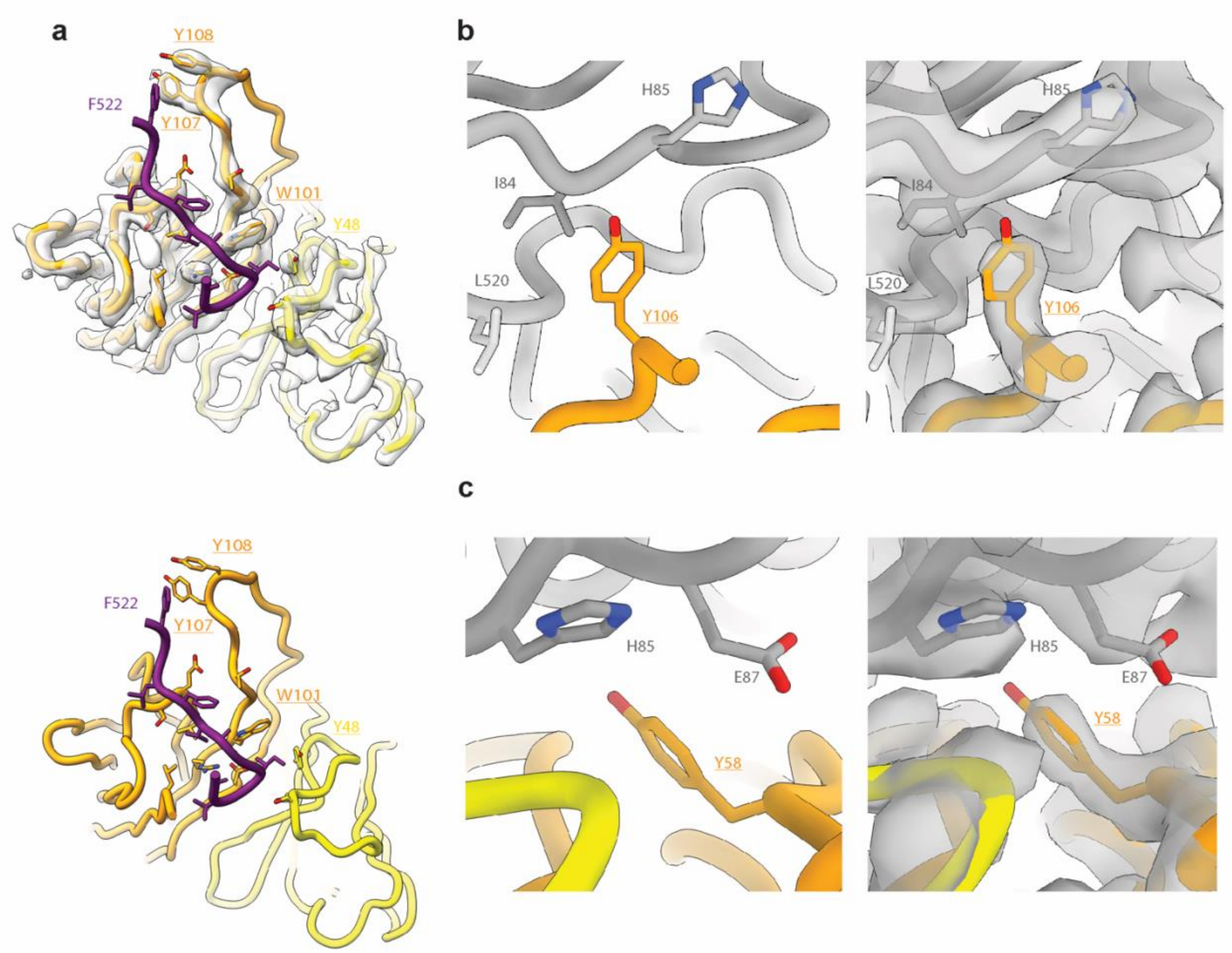


**Supplementary Figure 5. CryoEMPEM Polyclonal Residue Assignments**

A) FP3 map and model showing residue predictions (underlined residues) based on density information for FP interaction with HCDR3 and LFW2 aromatic residues. B) FP1 map and model showing residue predictions (underlined residues) based on density information for HCDR3 interaction with C-terminal FP residues and C1 region of gp120. C) FP1 map and model showing residue prediction (underlined residue) based on density information for HCDR2 interaction with C1 region of gp120.

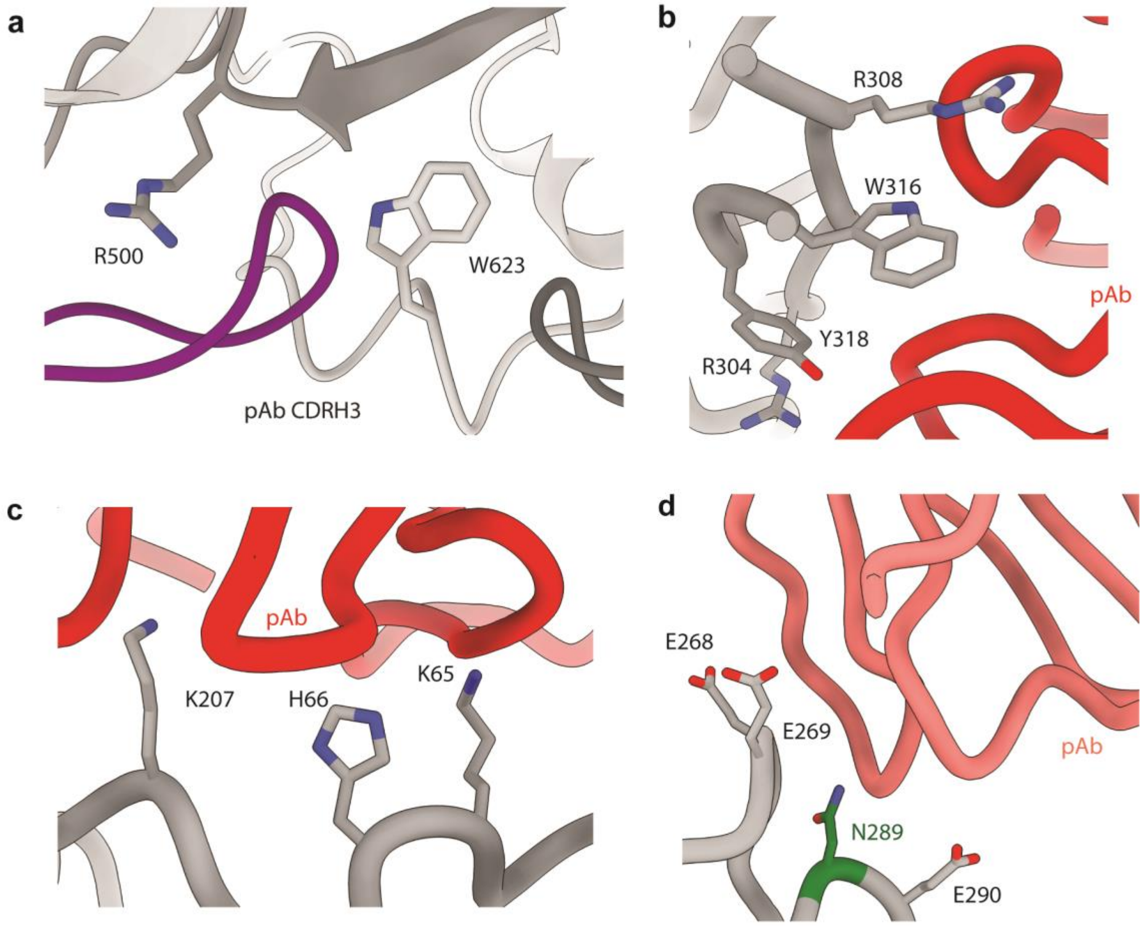


**Supplementary Figure 6. CryoEM Analysis of Off-target Responses**

A) Base response elucidated from Base-4 map shows predicted CDRH3 of pAb interaction with base of the trimer. B) IF response elucidated from IF-1 map shows antibody interactions with V3 region of trimer. C) IF response elucidated from IF-3 map shows antibody interactions with C1 region of trimer. D) N289 response shows antibody interaction with C2 region of trimer due to absence of N289 glycan.

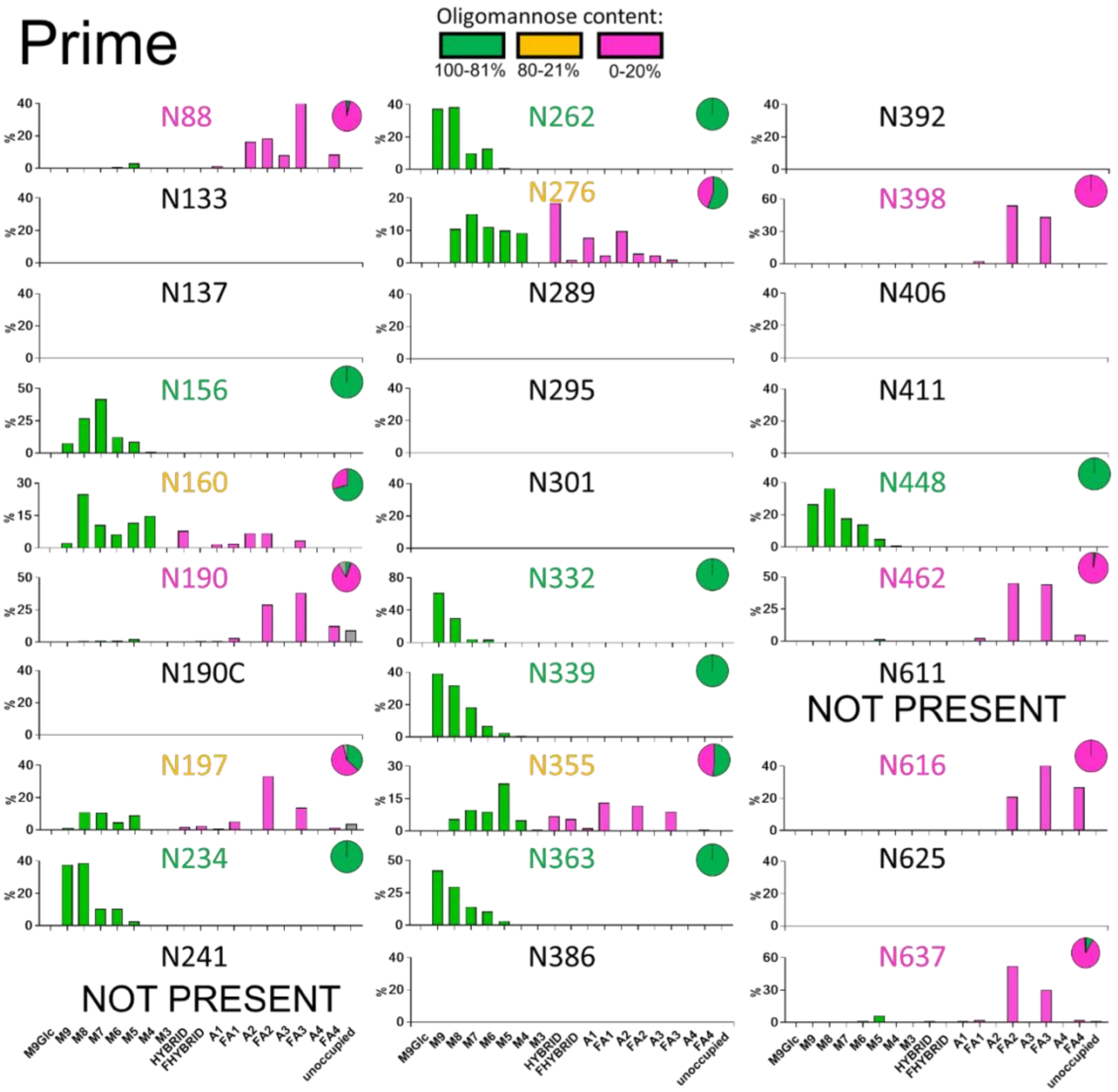


**Supplementary Figure 7. Site-specific analysis of the Prime immunogen.**

The intensity of different glycoforms at a single PNGS were compared and glycopeptides were categorized according to the number of mannose residues (green), hybrids that possess one unprocessed arm and one processed and also the number of processed branches and the presence/absence of fucose for complex-type glycans (magenta). The proportion of unoccupied asparagines at each site is colored grey. Pie charts summing the total oligomannose (M9-M4), complex and unoccupied are shown to the right of each site and the color of the lettering refers to the % oligomannose at each site, with green letters containing over 80% oligomannose, between 80% and 20% colored orange and less than 20% magenta.

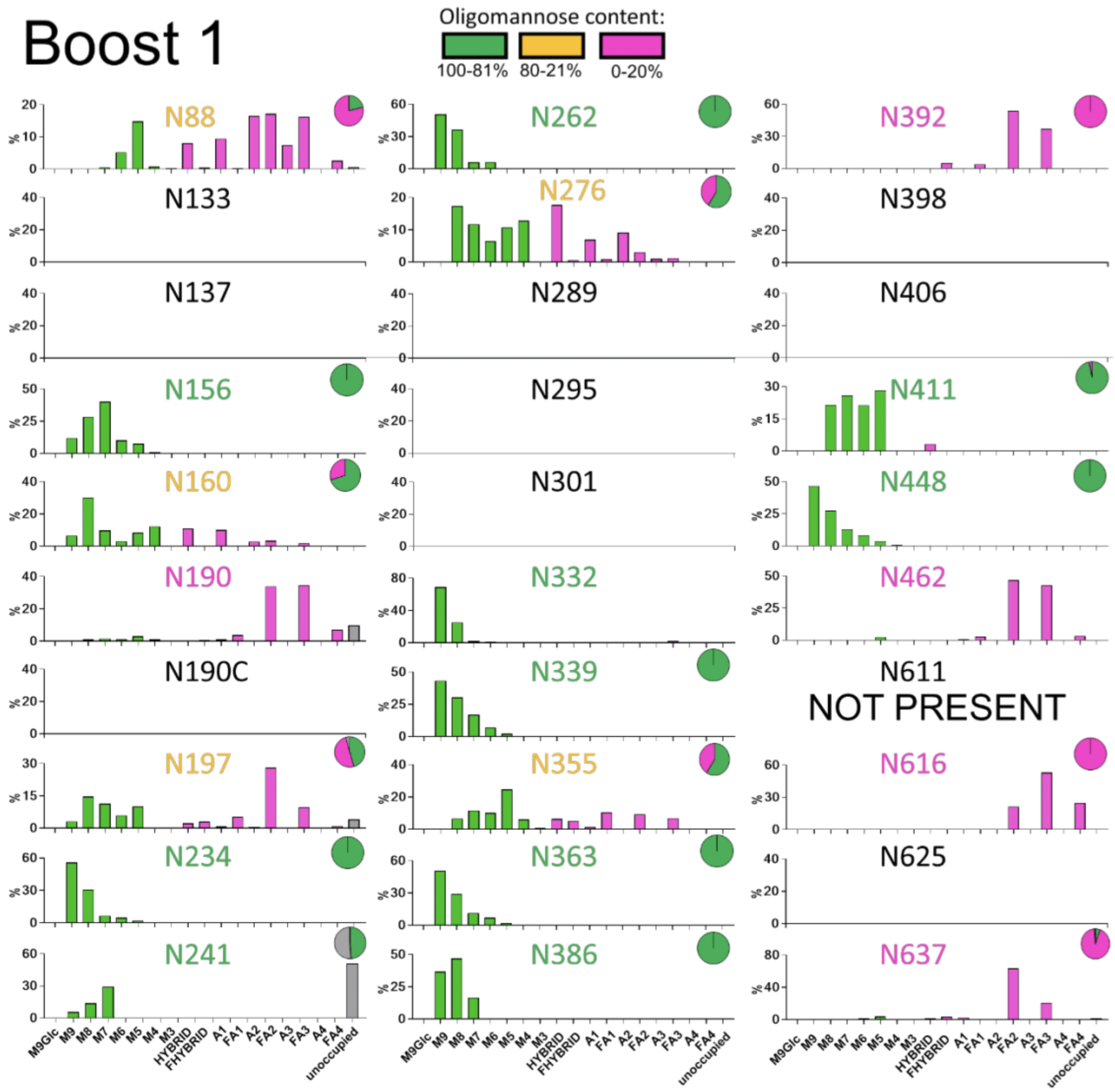


**Supplementary Figure 8. Site-specific analysis of the Boost#1 immunogen.**

Same as in Supplementary Figure 7

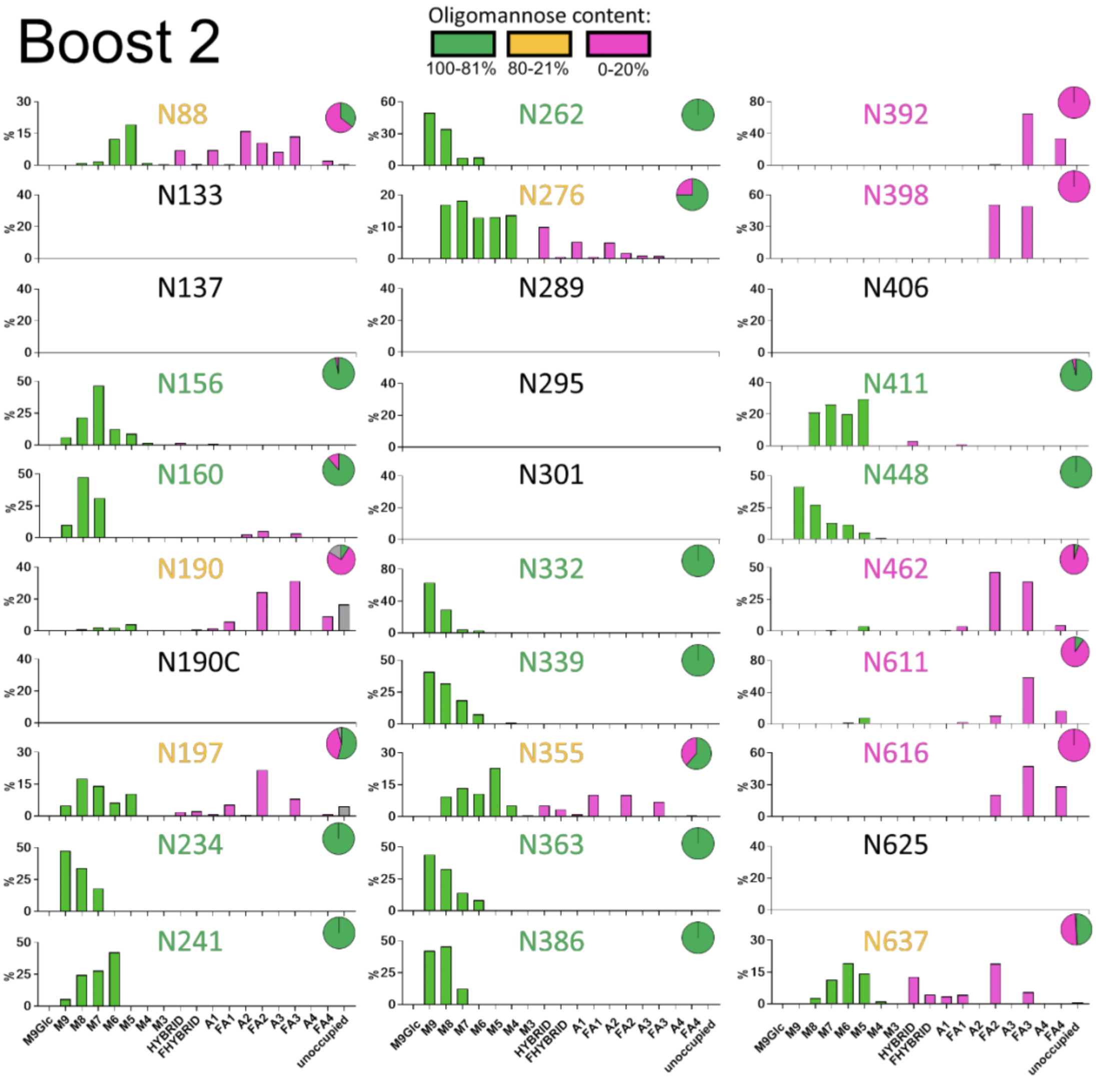


**Supplementary Figure 9. Site-specific analysis of the Boost#2 immunogen.**

Same as in Supplementary Figure 7

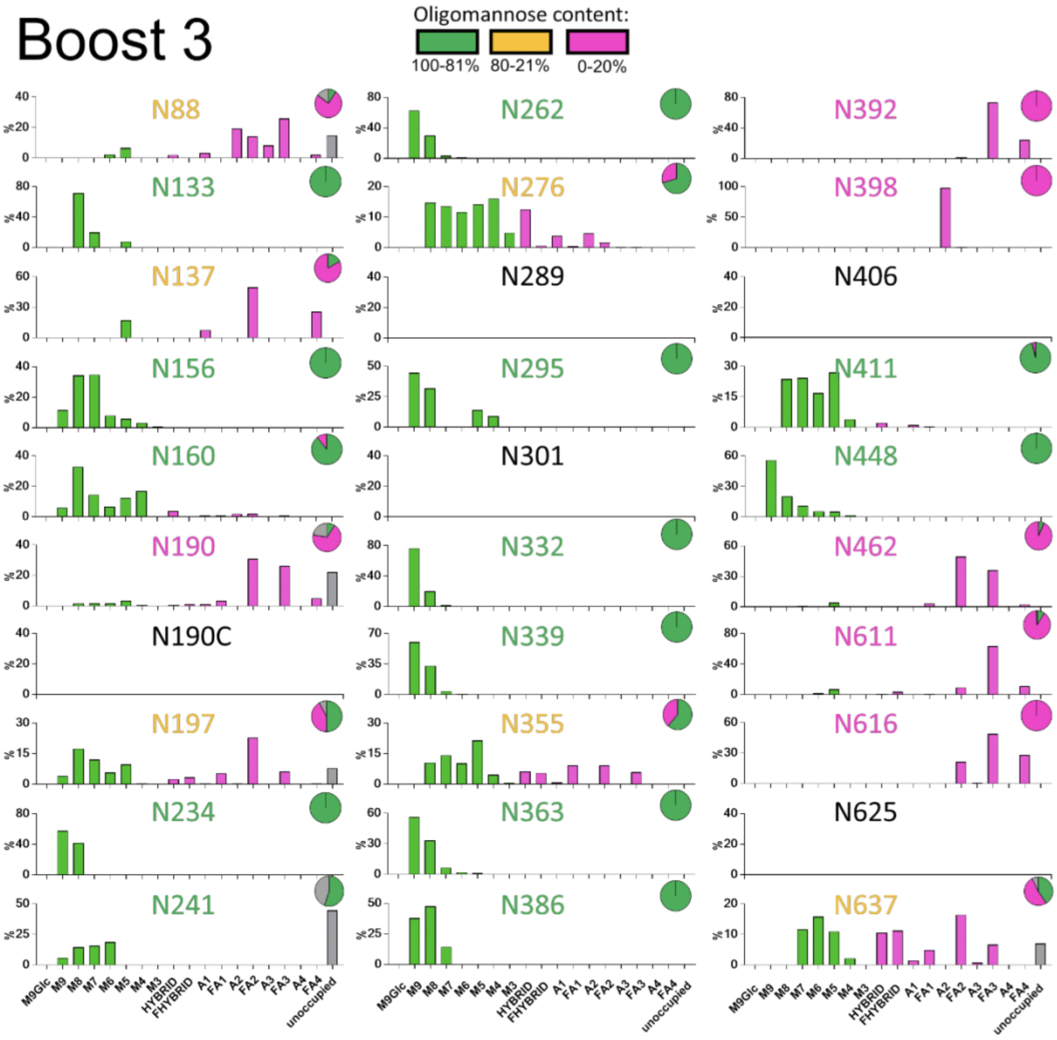


**Supplementary Figure 10. Site-specific analysis of the Boost#3 immunogen.**

Same as in Supplementary Figure 7

**Supplementary Table 1. BG505 pseudovirus neutralization data weeks 24, 26 and 42.**

| Study Group | Animal ID | Week | SVA-MLV | BG505 | BG505/ T332N | BG505/ T332N. N611A | BG505/ T332N. T465N | BG505/ T332N. 133aN+136aA |
|---|---|---|---|---|---|---|---|---|
| Control | 31943 | 24 | <20 | **2453** | **1409** | **1383** | **621** | **409** |
| Control | 31950 | 24 | <20 | **202** | **217** | **323** | **60** | **240** |
| Control | 32052 | 24 | <20 | **613** | **866** | **836** | **68** | **714** |
| Control | 32135 | 24 | <20 | **318** | **290** | **333** | <20 | **347** |
| Control | 32647 | 24 | <20 | <20 | <20 | <20 | <20 | <20 |
| Control | 32623 | 24 | <20 | **419** | **584** | **804** | **75** | **580** |
| Experimental | 32607 | 24 | <20 | **356** | **200** | **398** | **50** | **57** |
| Experimental | 32613 | 24 | <20 | <20 | <20 | **282** | <20 | <20 |
| Experimental | 32653 | 24 | <20 | 22 | 22 | **227** | <20 | 28 |
| Experimental | 32696 | 24 | <20 | 24 | **74** | **558** | **46** | 24 |
| Experimental | 32802 | 24 | <20 | <20 | 22 | **676** | <20 | <20 |
| Experimental | 32744 | 24 | <20 | 23 | <20 | **174** | <20 | <20 |
| Control | 31943 | 26 | <20 | **2069** | **1098** | **1000** | **643** | **199** |
| Control | 31950 | 26 | <20 | **104** | **134** | **159** | **50** | **137** |
| Control | 32052 | 26 | <20 | **305** | **314** | **412** | 28 | **365** |
| Control | 32135 | 26 | <20 | **197** | **174** | **210** | <20 | **231** |
| Control | 32647 | 26 | <20 | <20 | 28 | <20 | <20 | <20 |
| Control | 32623 | 26 | <20 | **273** | **288** | **527** | **57** | **325** |
| Experimental | 32607 | 26 | <20 | **486** | **312** | **483** | **125** | **77** |
| Experimental | 32613 | 26 | <20 | <20 | 21 | **256** | <20 | <20 |
| Experimental | 32653 | 26 | <20 | 39 | 33 | **183** | 21 | **50** |
| Experimental | 32696 | 26 | <20 | **40** | **91** | **468** | **47** | **50** |
| Experimental | 32802 | 26 | <20 | <20 | 22 | **465** | <20 | <20 |
| Experimental | 32744 | 26 | <20 | <20 | <20 | **94** | <20 | 22 |
| Control | 31943 | 42 | <20 | **1,663** | **1,134** | **1,209** | **394** | **393** |
| Control | 31950 | 42 | <20 | **1,179** | **1,199** | **1,269** | **397** | **1,190** |
| Control | 32052 | 42 | <20 | **315** | **302** | **281** | <20 | **322** |
| Control | 32135 | 42 | <20 | **4,286** | **4,366** | **5,063** | <20 | **5,070** |
| Control | 32647 | 42 | <20 | **49** | **88** | **73** | <20 | **74** |
| Control | 32623 | 42 | <20 | **1,743** | **1,737** | **1,970** | **521** | **1,777** |
| Experimental | 32607 | 42 | <20 | **1,823** | **1,361** | **2,288** | **314** | **638** |
| Experimental | 32613 | 42 | <20 | **81** | **68** | **3,589** | **47** | **50** |
| Experimental | 32653 | 42 | <20 | **125** | **80** | **534** | <20 | <20 |
| Experimental | 32696 | 42 | <20 | **224** | **234** | **643** | **42** | **37** |
| Experimental | 32802 | 42 | <20 | **101** | **98** | **4,356** | **48** | **82** |
| Experimental | 32744 | 42 | <20 | **78** | **81** | **475** | **44** | **70** |

Values are the serum dilution at which relative luminescence units (RLUs) were reduced 50% compared to virus control wells (no test sample)

**Supplementary Table 2. FP-sensitive pseudovirus panel neutralization data weeks 24, 26, and 42.**

| Study Group# | Animal ID | Week | 25710-2.43 | 3988.25 | 0077.V1.C16 | CNE19 | CNE56 | KER2008.vrc12 | Q23.17 | 286_36 | BL01.DG |
|---|---|---|---|---|---|---|---|---|---|---|---|
| Control | 31943 | 24 | <20 | <20 | <20 | **214** | <20 | <20 | <20 | <20 | <20 |
| Control | 31950 | 24 | <20 | 26 | <20 | 24 | <20 | <20 | <20 | <20 | <20 |
| Control | 32052 | 24 | <20 | 26 | <20 | <20 | <20 | <20 | <20 | <20 | <20 |
| Control | 32135 | 24 | <20 | 32 | <20 | 34 | <20 | <20 | <20 | 24 | <20 |
| Control | 32647 | 24 | <20 | 31 | <20 | <20 | <20 | <20 | <20 | <20 | <20 |
| Control | 32623 | 24 | <20 | <20 | <20 | <20 | <20 | <20 | <20 | <20 | <20 |
| Experimental | 32607 | 24 | <20 | <20 | <20 | <20 | <20 | <20 | <20 | <20 | <20 |
| Experimental | 32613 | 24 | <20 | <20 | <20 | 32 | <20 | <20 | <20 | <20 | <20 |
| Experimental | 32653 | 24 | <20 | 34 | <20 | <20 | <20 | <20 | <20 | <20 | <20 |
| Experimental | 32696 | 24 | <20 | 24 | <20 | 20 | <20 | <20 | <20 | <20 | <20 |
| Experimental | 32802 | 24 | <20 | <20 | <20 | <20 | <20 | <20 | <20 | <20 | <20 |
| Experimental | 32744 | 24 | <20 | 26 | <20 | <20 | <20 | <20 | <20 | <20 | <20 |
| Control | 31943 | 26 | <20 | <20 | <20 | **136** | <20 | <20 | <20 | <20 | <20 |
| Control | 31950 | 26 | <20 | 25 | <20 | 27 | <20 | <20 | <20 | <20 | <20 |
| Control | 32052 | 26 | <20 | <20 | <20 | <20 | <20 | <20 | <20 | <20 | <20 |
| Control | 32135 | 26 | <20 | 31 | <20 | 21 | <20 | <20 | <20 | 21 | <20 |
| Control | 32647 | 26 | <20 | **55** | <20 | 22 | <20 | <20 | <20 | <20 | 36 |
| Control | 32623 | 26 | <20 | <20 | <20 | <20 | <20 | <20 | <20 | <20 | <20 |
| Experimental | 32607 | 26 | <20 | 22 | <20 | <20 | <20 | <20 | <20 | <20 | <20 |
| Experimental | 32613 | 26 | <20 | 21 | <20 | 26 | <20 | <20 | <20 | <20 | <20 |
| Experimental | 32653 | 26 | <20 | **42** | <20 | 32 | <20 | <20 | 21 | 23 | <20 |
| Experimental | 32696 | 26 | 22 | 39 | <20 | 30 | <20 | <20 | 27 | <20 | **41** |
| Experimental | 32802 | 26 | <20 | <20 | <20 | <20 | <20 | <20 | <20 | <20 | <20 |
| Experimental | 32744 | 26 | <20 | 29 | <20 | 22 | <20 | <20 | <20 | <20 | <20 |
| Control | 31943 | 42 | <20 | <20 | <20 | <20 | <20 | <20 | <20 | <20 | <20 |
| Control | 31950 | 42 | <20 | <20 | <20 | <20 | <20 | <20 | <20 | <20 | <20 |
| Control | 32052 | 42 | <20 | <20 | <20 | <20 | <20 | <20 | <20 | <20 | <20 |
| Control | 32135 | 42 | <20 | <20 | <20 | <20 | <20 | <20 | <20 | <20 | <20 |
| Control | 32647 | 42 | <20 | <20 | <20 | <20 | <20 | <20 | <20 | <20 | <20 |
| Control | 32623 | 42 | <20 | <20 | <20 | <20 | <20 | <20 | <20 | <20 | <20 |
| Experimental | 32607 | 42 | <20 | <20 | <20 | <20 | <20 | <20 | <20 | <20 | <20 |
| Experimental | 32613 | 42 | **53** | **42** | <20 | **148** | <20 | <20 | <20 | <20 | <20 |
| Experimental | 32653 | 42 | **<20** | **27** | <20 | **<20** | <20 | <20 | <20 | <20 | <20 |
| Experimental | 32696 | 42 | <20 | <20 | nd | <20 | nd | <20 | <20 | nd | <20 |
| Experimental | 32802 | 42 | <20 | <20 | <20 | **34** | <20 | <20 | <20 | <20 | <20 |
| Experimental | 32744 | 42 | **30** | **32** | <20 | **37** | <20 | <20 | <20 | <20 | <20 |

Values are the serum dilution at which relative luminescence units (RLUs) were reduced 50% compared to virus control wells (no test sample)

## Supplementary Table 3. Global panel pseudovirus neutralization data.

| | | | ID50 (dilution) in TZM-bl cells | | | | | | | | | |
|---|---|---|---|---|---|---|---|---|---|---|---|---|
| Study Group# | Animal ID | Week | CEO217 | CE1176 | 25710 | CH119 | X2278 | TRO11 | BJOX2000 | CNE55 | X1632 | 246F3 |
| Crtl | 31943 | 42 | <10 | <10 | <10 | <10 | <10 | <10 | <10 | <10 | <10 | <10 |
| Crtl | 31950 | 42 | <10 | <10 | <10 | <10 | <10 | <10 | <10 | <10 | <10 | <10 |
| Crtl | 32052 | 42 | <10 | <10 | <10 | <10 | <10 | <10 | <10 | <10 | <10 | <10 |
| Crtl | 32135 | 42 | <10 | <10 | <10 | <10 | <10 | <10 | <10 | <10 | <10 | <10 |
| Crtl | 32647 | 42 | <10 | <10 | <10 | 13 | <10 | <10 | <10 | <10 | <10 | <10 |
| Crtl | 32623 | 42 | <10 | <10 | <10 | <10 | <10 | <10 | <10 | <10 | <10 | <10 |
| Expt | 32607 | 42 | <10 | <10 | 59 | 19 | <10 | <10 | <10 | <10 | <10 | <10 |
| Expt | 32613 | 42 | <10 | 13 | 13 | <10 | <10 | <10 | <10 | <10 | <10 | <10 |
| Expt | 32653 | 42 | <10 | <10 | <10 | <10 | <10 | <10 | <10 | <10 | <10 | <10 |
| Expt | 32696 | 42 | <10 | <10 | <10 | <10 | <10 | <10 | <10 | <10 | <10 | <10 |
| Expt | 32802 | 42 | <10 | <10 | 15 | <10 | <10 | <10 | <10 | <10 | 12 | <10 |
| Expt | 32744 | 42 | <10 | 13 | 75 | <10 | <10 | <10 | <10 | <10 | 15 | <10 |

Values are the serum dilution at which relative luminescence units (RLUs) were reduced 50% compared to virus control wells (no test sample)

**Supplementary Table 4. Immunogen mutations relative to BG505 SOSIP.v5.2**

| Immunogen | Mutations relative to BG505 SOSIP.v5.2 |
|---|---|
| Prime | M271I-F288L-T290E-P291S-N611Q |
| Boost#1 | P240T-S241N-M271I-F288L-T290E-P291S-N611Q |
| Boost#2 | P240T-S241N-M271I-F288L-T290E-P291S-S613T |
| Boost#3 | H85V-K229N-P240T-S241N-M271I-F288L-T290E-P291S-S613T |

**Supplementary Table 5. FACS antibody panel.**

| Marker | Clone | Flurophore |
|---|---|---|
| Viability | n/a | APCe780 |
| CD20 | 2H7 | Ax488 |
| CD4 | OKT-4 | APCe780 |
| CD8a | RPA-T8 | APCe780 |
| CD16 | ebioCB16 | APCe780 |
| IgG | G18-145 | AF700 |
| IgM | G20-127 | PerCP-Cy5.5 |
| CD71 | L01.1 | PE-CF594 |
| CD38 | OKT | PE |
